# Empirical Evidence for Impacts of Internal Migration on Vegetation Dynamics in China from 1982 to 2000

**DOI:** 10.3390/s8085069

**Published:** 2008-08-27

**Authors:** Conghe Song, Jackson W. Lord, Liming Zhou, Jingfeng Xiao

**Affiliations:** 1 Department of Geography, University of North Carolina, Chapel Hill, NC 27599, USA; Email: csong@email.unc.edu; 2 Department of Geography, University of North Carolina, Chapel Hill, NC 27599, USA; Email: jacksonlord@gmail.com; 3 Department of Earth and Atmospheric Sciences, Georgia Institute of Technology, Atlanta, GA, 30332, USA; Email: lmzhou@eas.gatech.edu; 4 Department of Meteorology, Pennsylvania State University, University Park, State College, PA 16802, USA; Email: jing@psu.edu

**Keywords:** Rural-to-Urban Migration, NDVI, Human-Environment Interactions, China

## Abstract

Migration is one of the major socio-economic characteristics of China since the country adopted the policy of economic reform in late 1970s. Many studies have been dedicated to understand why and how people move, and the consequences of their welfare. The purpose of this study is to investigate the environmental impacts of the large scale movement of population in China. We analyzed the trend in the Normalized Difference Vegetation Index (NDVI) from the Advanced Very High Resolution Radiometer (AVHRR) along with China migration data from the 1 percent national survey during 1982-1987, the 4^th^ national census during 1985-1990 and the 5^th^ national census during1995∼2000. We found that the internal migration in China has a statistically significant negative impact on vegetation growth at the provincial scale from 1982 to 2000 even though the overall vegetation abundance increased in China. The impact from migration (R^2^=0.47, P=0.0001) on vegetation dynamics is the second strongest as among the factors considered, including changes in annual mean air temperature (R^2^=0.50, P=0.0001) and annual total precipitation (R^2^=0.30, P=0.0049) and gross domestic production (R^2^= 0.25, P=0.0102). The negative statistical relationship between the rate of increase in total migration and the change in vegetation abundance is stronger (R^2^=0.56, P=0.0000) after controlling for the effects of changes in temperature and precipitation. In-migration dominates the impacts of migration on vegetation dynamics. Therefore, it is important for policy makers in China to take the impacts of migration on vegetation growth into account while making policies aiming at sustainable human-environment relations.

## 1. Introduction

Since the adoption of the policy of economic reform in China in late 1970s, a large amount of concealed surplus labor force in the old communal system in the rural area was released (Zhao, 1999; Lu et al, 2005). Given the government relaxation in limiting rural to urban migration and the increasing income gaps between the cities and rural areas, millions of people from the rural areas migrate to the cities to seek better economic fortunes every year. The total migration, including intra-, in- and out-provincial migration, from 1982∼1987 to 1995∼2000 increased from 36 million/year to 165 million/year (Li, 1994; Fan, 2005a). The scale and number of human migration in China is unprecedented (Zhao, 1999; Liang, 2001). The vast resource of cheap migrant labor helps sustain the competitiveness of labor intensive goods manufactured in China. Current literature on migration in China primarily focused on why and how migrants move from place to place, and the consequences of their welfare (Zhao, 1999; Liang, 2001; Liang and Ma, 2004; Fan, 2005a). Little is known about its environmental effects. Migration has two potential contrasting impacts on vegetation. On the one hand, the enormous exodus of people from rural areas allows regrowth of vegetation and reduces deforestation; on the other hand, huge rural-to-urban migration provided an almost unlimited labor force for construction and the expansion of manufacturing industry, causing rapid urban sprawl and loss of vegetation (Heilig, 1997). Urban area in China increased 25 percent from 1990 to 2000 based on satellite observations (Liu et al., 2005). In the southern coastal regions, the expansion of urban areas can be as much as 30 percent per year (Ji, et al., 2001). China lost approximately 500,000 ha/year of agricultural land to development in the 1990s (Smil, 1999). However, several other recent studies found increased vegetation activity in China (Fang et al., 1999; Xiao and Moody, 2004). The purpose of this study is to understand the environmental impacts of the large scale rural-to-urban migration, which has profound implications on the already fragile human-environment connections in China. Rural-to-urban migration is an international phenomenon, particularly in the developing countries, such as Viet Nam (Dang et al., 1997) and Brazil (Perz, 2003). Understanding the impacts of migration on the environment in China has global implications.

## 2. Data and Methods

Our analysis is based on two sets of data: migration data from census and a vegetation index from remote sensing. We used China migration data at the provincial scale published in the literature for 1982-1987 (1 percent national sample survey), 1985-1990 (4^th^ national census), and 1995-2000 (5^th^ national census), as shown in Table 1 (Li, 1994; Fan, 2005; China Data Center, 2006). For convenience, we will refer the three time intervals as τ_1_, τ_2_, and τ_3_, respectively. The environmental settings for the three municipal cities, Beijing, Shanghai and Tianjin, are significantly different from other provinces or autonomous regions. We excluded these cities in the analysis. In addition, Taiwan, Macau, Hongkong, and Xizang were not included in the analysis due to lack of data in the literature. It is well known that the definition of migration is not consistent during the three periods. A migrant before and during the 4^th^ census was defined as one who left his/her *hukou* (household registration) location for more than one year. The temporal criterion was revised from one year to six months in the 5^th^ census. It is difficult to assess the effect of the change in the definition on the total migration assessment (Fan, 2005b). However, it is reasonable to assume that the difference in the definition of migration causes systematic errors which would not significantly alter the results of subsequent statistical analysis. There are three types of migration with regard to its direction and distance: intra-, in-, and out-provincial migration. Due to the differences in the areas and population among the provinces, the total migration do not compare well. We normalized the increase in migration to become the rate of increase in migration for better comparison among provinces as in the following:

(1) $$\Delta {\text{M}}_{\text{ij}}=\frac{{\text{M}}_{\text{i}}-{\text{M}}_{j}}{{\text{M}}_{\text{j}}},$$

where ΔM_ij_ (j<i) is the rate of change in migration from τ_j_ to τ_i_, and M_i_ and M_j_ is the number of migrants in a province at time τ_i_ and _j_. ΔM_ij_ is calculated for intra-, in-, out-migration and total migration, respectively.

The abundance of vegetation is quantified by the Normalized Difference Vegetation Index (NDVI), which is a standard measure of the abundance of active green vegetation with satellite observations. NDVI varies within [-1, 1] with a higher NDVI indicating more abundance of green vegetation. It is calculated based on the reflectance measured in the red and near-infrared spectra as

(2) $$\text{NDVI}=\frac{\rho_{\text{NIR}}-\rho_{\text{RED}}}{\rho_{\text{NIR}}+\rho_{\text{RED}}},$$

where ρ_NIR_ and ρ_RED_ are reflectance in the near-infrared and red bands, respectively.

**Figure 1.**
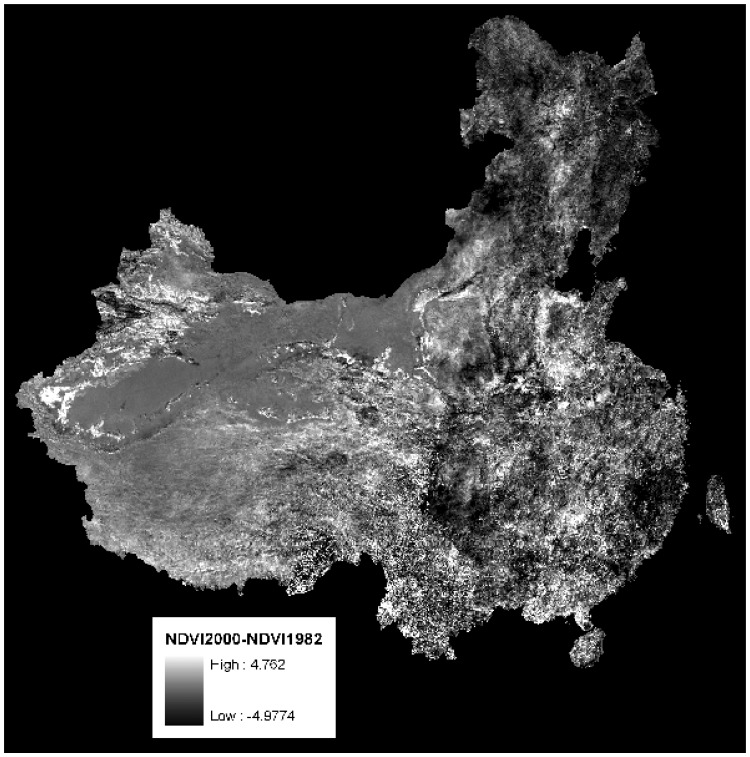
The difference between the average mean annual total NDVI (AMATN) from 1982∼1987 to 1995∼2000. To get AMATN, we first calculate the annual total NDVI (ATN) for each pixel from the 24 NDVI images, and then we take the mean of the ATN (MATN) for all pixels within a province. We took an average for MATN (AMATN) for all years within 1982∼1987 or 1995∼2000. There are a lot of decreases in NDVI in the eastern part of China where most of the migrants go.

The remotely sensed data used in this study is the continuous measurements of time series NDVI from the Advanced Very High Resolution Radiometer (AVHRR) on board the NOAA-series satellites from 1982 to 2000 with a spatial resolution of 8×8 km (Tucker, et al., 2005). AVHRR provides daily NDVI measurements for the entire globe. However, NDVI can be contaminated by the aerosols and the clouds in the atmosphere. The dataset used in this study is a 15-day composite NDVI, which takes the maximum NDVI from the 15 daily values for each pixel to minimize the contamination from the atmosphere. Therefore, there are twenty four AVHRR NDVI images for China each year. We use the annual total NDVI (ATN) as indicator for vegetation abundance in this study. Due to the fact that each census or 1 percent sample survey spanned six years, we took the mean ATN (MATN) during the same six years as the measure of vegetation amount so that the measurement of vegetation abundance corresponds to the same period of time over which the migration data were collected. NDVI for water pixels was set to -1. Pixels that remain water for the entire year is excluded from the analysis, i.e. pixels with ATN being -24 were not used because their NDVI is not influenced by migration. After the 15-day composite, some pixels remain contaminated by clouds. These pixels were flagged in the NDVI dataset. Single missing value due to cloud contamination was filled with the average of the two NDVI values from the same pixel that were immediately before and after the missing one in time. If there were two missing values next to each other in time, we replaced the first missing value with the NDVI that is immediately before and the second one with that immediate after the missing value. If a pixel had three or more missing values within a year, the NDVI of the pixel for that year was considered missing, and MATN was calculated with the remaining data points during the 6 years. We did not perform any spatial interpolation to fill missing values as the process might change the spatial pattern of vegetation, which is critical to our analysis. We then converted the MATN to a point coverage using ArcGIS version 9.1 using the longitude and latitude at the center of the pixel as the coordinates for the points. The point coverages were overlayed on the 1982 provincial polygons. For the convenience of comparison through time, we merged Hainan province with Guangdong and Chongqiang municipal city with Sichuan province as they were separated out from the corresponding provinces in 1988 and 1997, respectively. Otherwise, the data from these provinces could not be compared through time. We did a point in polygon analysis and calculated the average of MATN (AMATN) for all points within a province for each census period. Unlike migration, changes in NDVI are comparable among provinces without normalization. We evaluated the change in vegetation abundance in each province as in the following:

(3) $$\Delta {\text{V}}_{\text{ij}}={\text{AMATN}}_{i}-{\text{AMATN}}_{j},$$

where ΔV_ij_ (j<i) is the change in vegetation abundance from τ_j_ to τ_i_, and AMATN_i_ and AMATN_j_ are the provincial Average of the Mean Annual Total NDVI during τ_i_ and τ_j_, respectively. ΔV_31_ is the change in AMATN from τ_1_ to τ_3_ as shown in Figure 1. We did not present results for the analysis from τ_1_ to τ_2_ due to the overlap and short interval in time.

Growth of vegetation without human disturbance is primarily determined by temperature and precipitation (Lieth, 1972). Due to the trend of global warming, changes in temperature and precipitation can also alter vegetation dynamics in China. Therefore, we also analyze the relationship between change in temperature and precipitation and the change in vegetation abundance. The annual mean temperature and annual total precipitation for each province in China were obtained from the global monthly climatology dataset available at Oak Ridge National Laboratory (http://www.daac.ornl.gov). We did not normalize the changes in temperature and precipitation as we did for migration as they are comparable across the provinces. The change in temperature is calculated as ΔT_ij_=T_i_-T_j_, where j<i, and T_i_ and T_j_ are the provincial mean annual temperature during τ_i_ and τ_j_, respectively, and the change in precipitation is calculated similarly as ΔP_ij_=P_i_-P_j_. Due to the fact that climate change is related to human activities (Zhou et al., 2004; Kaufmann et al., 2007), we need to remove these effect in the climate data in order to fully understand the impacts from human activities. Our data show there is a statistically significant relationship between migration and changes in temperature and precipitation. Therefore, we first regressed ΔT_ij_ and ΔP_ij_ with ΔM_ij_, separately, and obtain the residuals from the regressions, _r_ΔT_ij_ and _r_ΔP_ij_. These residuals of change in temperature and precipitation would not contain the effect from migration. We then did a multiple regression of ΔV_ij_ with _r_ΔT_ij_ and _r_ΔP_ij_, and obtain the residuals, _r_ΔV_ij_, from which the impacts of changes in temperature and precipitation are removed, while the impact from changes in migration remains. We finally did a regression between _r_ΔV_ij_ with ΔM_ij_ to evaluate the impact of migration on vegetation dynamics controlled for effect of the changes in temperature and precipitation.

In addition to temperature and precipitation, we also studied the impact of the change in gross primary production (GDP) (China Compendium of Statistics, 2005) on vegetation abundance to account for possible impact from other sectors of the economy. Similar to migration, change in GDP was normalized to become rate of increase in GDP from τ_j_ to τ_i_ (ΔGDP_ij_).

## 3. Results and Discussions

The three data points, 1982∼1987 (τ_1_), 1985∼1990 (τ_2_), and 1995∼2000 (τ_3_), allow us to examine the impacts of migration on vegetation dynamics between three periods. However, the first two data points have a two-year overlap, thus results between these two periods were not included here. We analyzed the relationships between ΔV_ij_ and ΔM_ij_ for intra-, in-, and out-migration as well as for the total migration during [τ_1_, τ_3_] and [τ_2_, τ_3_]. Regression results are given in Table 2, indicating that all forms of migration negatively influences vegetation abundance. The influence of in-migration on vegetation abundance is statistically significant for both periods, and dominates the effect of migration. In-migrants are pulled by existence of better economic opportunities. Migrant workers often take low-skill labor intensive manufacturing jobs in the cities, thus more in-migrants are indicative of industrial expansion, which is usually associated with urban sprawl and causes dramatic decrease in NDVI. Though the influences from intra- and out-provincial migration are not statistically significant either for [τ_1_, τ_3_] or for [τ_2_, τ_3_], the negative impacts are increasing. Intra-provincial migration has similar effect on vegetation as in-migration, but to a much smaller extend. Net economic gain is the driving factor for rural-to-urban migration. In general, there is a high cost associated with migration from one province to another. There must be a greater economic return at the destination for in-migrants. A greater vigor of industrial growth is needed to attract people from another province than attracting people within the province. Therefore, a greater deal of urban expansion is associated with in-migration than intra-provincial migration, leading to stronger negative impact on vegetation growth for in-migration compared with intra-provincial migration. Contrary to our expectation, out-migration also negatively influences vegetation abundance, though not statistically significant. We originally hypothesized that reduction of population in the rural areas allows the regrowth of vegetation. However, remittance sending back from out-migrants may expedite land-cover/land-use change in the rural area, reducing vegetation growth. Based on a recent trip to the rural areas in Anhui province, a major out-migration province, the first author of this study observed that a large proportion of households with people working away built a new house with the money earned. Therefore, the negative effect on vegetation from out-migration outweighs the positive effect at the provincial scale.

**Figure 2.**
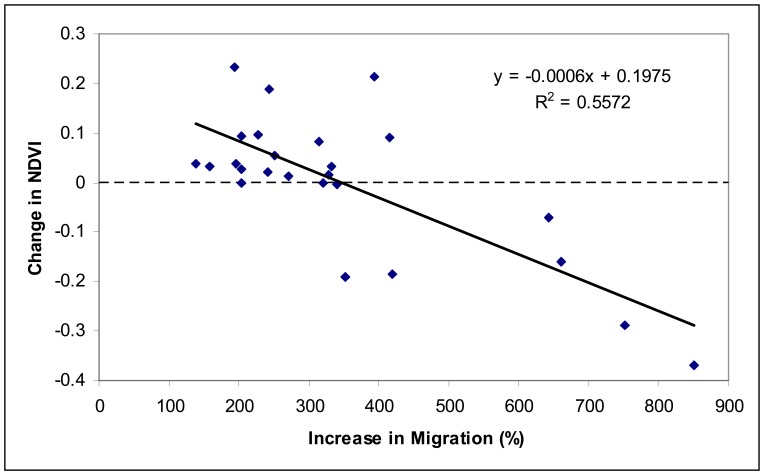
Relationship between change in NDVI and rate increase in total migration, including intra-, in- and out-migration, from 1982 to 2000 for each province listed in Table 1 after controlling the effects from changes in temperature and precipitation.

The statistical results in Table 2 contain the confounding effects from other factors, particularly changes in temperature, precipitation, as well as other economic activities. Therefore, we evaluated the percent increase in vegetation abundance with temperature, precipitation and GDP (Table 3). The relationship between change in vegetation abundance from τ_1_ to τ_3_ (ΔV_31_) is also statistically significant with rate of increase in gross domestic production (ΔGDP_31_), but its R^2^ is lower compared to changes in temperature and precipitation as well as the rate of increase in migration. GDP reflects the economic activities in all sectors of the economy, many of which are not directly influenced by rural-to-urban migration though migrant related manufacture is a significant component of GDP in China. Therefore, GDP should be significantly related to decrease in vegetation, but to a less degree compared to migration. Figure 2 shows the negative relationship between the rate of increase in total migration from τ_1_ to τ_3_ and change in AMATN during the same period for each province as listed in Table 1 after controlling the effects from changes in temperature and precipitation. The relationship is stronger compared to that in Table 2, indicating climate change obscured the impacts of migration on vegetation dynamics.

Both changes in temperature and precipitation significantly influence change in vegetation abundance. Increase in temperature increases vegetation abundance. This agrees with satellite observations in other parts of the world as a result of global warming (Myneni et al., 1997; Zhou et al., 2003). However, the change in vegetation abundance is negatively correlated with changes in precipitation. Further analysis found that the change in precipitation is negatively correlated with change in temperature in China from τ_1_ to τ_3_ (Figure 2). The R^2^ between ΔT_ij_ and ΔV_ij_ is much higher than that between ΔP_ij_ and ΔV_ij_ in Table 3, thus temperature dominated the effect of climate change on vegetation dynamics during this time in China.

Though the negative relationship is statistically significant and strong between the change in vegetation abundance and the rate of increase in migration in Figure 2, the overall NDVI in China increased during 1982∼2000 (Fang et al., 2004). Figure 2 does not contradict the finding as the change in vegetation abundance for most of the provinces are positive. Despite the rapid urbanization and many other environmental problems created by the fast economic growth (Liu et al., 2005), a positive feedback from the economic growth is the increased investment in environmental projects (Nei, 2005), which would not be affordable otherwise. As a result, China's forest cover increased dramatically from 12.0 percent in 1982 percent to 18.2 percent in 2003 (Zhang and Song, 2006). China returned over 24 million hectares of low productivity agricultural land to forest since 1999, increasing forest cover by 2 percentage points (People's Daily, August 26, 2007). Given that there are many other factors that may influence vegetation growth in China, it is impressive that total migration alone explained 55 percent of the variation in the change in NDVI at provincial scale after controlling for temperature and precipitation effects. Therefore, migration should be an important factor in making environmental policies, such as those aiming at carbon sequestration via increased vegetation growth.

## 4. Conclusions

There are statistically significant empirical evidences that large scale internal migration in China from 1982 to 2000 negatively influences vegetation growth in China based on satellite observations of vegetation abundance and migration data at the provincial scale. The statistical relationship is stronger after controlling for the effect of changes in annual mean air temperature and annual total precipitation. All three forms of migration, in-, intra- and out-provincial migration have negative impacts on vegetation growth. In-provincial migration dominates the impacts. Though neither intra- nor out-provincial migration has a statistically significant impact on vegetation dynamics, their influences are increasing with time. It is important for policy makers in China to take the impacts of migration on vegetation growth into account while making policies aiming at sustainable human-environment relations.

## Figures and Tables

**Figure 3.**
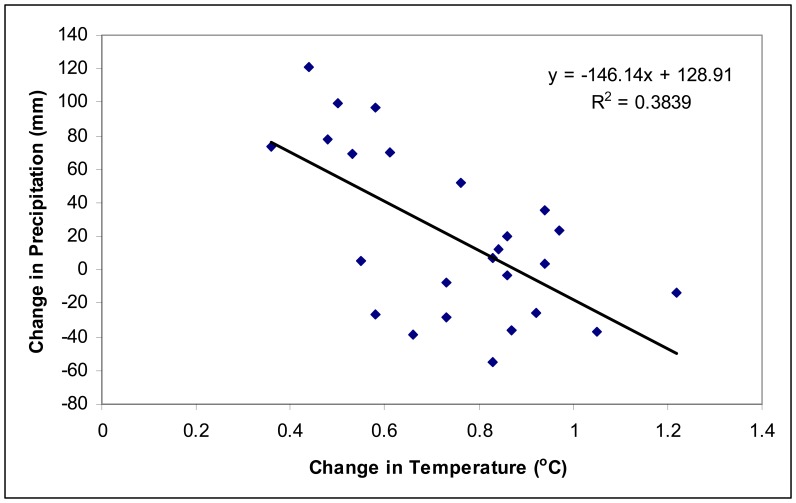
Relationship between changes in temperature and precipitation from 1982∼1987 to 1995∼2000. The negative relationship explains the opposite relationship of changes in temperature and precipitation with vegetation abundance in Table 3.

**Table 1.** The number of migrants, including intra-, in- and out-provincial migration during 1982∼1987 (τ_1_), 1985∼1990 (τ_2_) and 1995∼2000 (τ_3_) (Unit: 10^3^ persons). The three municipal cities (Beijing, Shanghai, and Tianjing) are not included in the analysis as the process of Land-Cover/Land-Use Change for these cities are very different from other provinces and autonomous regions. We do not have data for Taiwan, Hongkong, Macao, and Xizang. We merged the data from Hainan with Guangdong, and Chongqing with Sichuan for data consistency.

Province	*τ*_1_			*τ*_2_			*τ*_3_		
		
	Intra	Out	In	Intra	Out	In	Intra	Out	In
		
Hebei	942	371	594	819	653	524	3,951	872	769
Shanxi	800	185	168	632	220	310	3,053	333	382
Neimenggu	572	207	167	582	305	257	3,280	441	325
Liaoning	975	231	314	884	296	541	5,437	380	754
Jilin	918	238	168	605	351	234	2,641	529	254
Heilongjiang	877	449	192	1,063	613	368	3,382	940	301
Jiangsu	1,352	324	476	1,198	629	799	6,563	1,240	1,907
Zhejiang	795	239	124	818	648	343	4,910	968	2,714
Anhui	856	248	164	877	538	340	3,328	2,892	313
Fujian	469	112	92	732	240	255	3,766	624	1,346
Jiangxi	541	149	102	743	297	229	3,112	2,680	235
Shandong	1,507	339	544	1,188	531	607	6,435	878	903
Henan	921	326	269	1,254	597	484	4,724	2,306	468
Hubei	1,633	225	276	1,099	348	435	5,095	2,209	605
Hunan	1,226	376	220	1,308	532	275	4,047	3,260	362
Guangdong	2,024	140	268	2,800	357	1,401	10,835	568	11,718
Guangxi	675	213	60	891	590	144	2,806	1,838	287
Sichuan	3,294	471	366	2,368	1,330	443	8,351	5,091	660
Guizhou	556	123	117	467	317	193	2,007	1,231	261
Yunnan	647	184	95	739	280	250	2,707	397	731
Shaanxi	784	284	222	713	365	312	1,939	716	420
Gansu	406	189	93	453	282	198	1,330	555	203
Qinghai	63	103	29	152	102	115	3,98	120	76
Ningxia	92	51	92	123	57	92	481	87	129
Xinjiang	354	238	200	364	280	344	1,419	216	1,142

**Table 2.** Regression analysis between change in NDVI and rate of change in migration from 1982∼1987 to 1995∼2000 and from 1985∼1990 to 1995∼2000: ΔV_ij_ = b_0_ + b_1_ΔM_ij_.

Independent Variable	b0	b1	R^2^	P-value
1982∼1987 - 1995∼2000				

Total Migration	0.30211	-0.05801	0.4743	0.0001
In-Migration	0.14768	-0.01023	0.3589	0.0016
Out-Migration	0.14499	-0.01045	0.0848	0.1579
Intra-Migration	0.22401	-0.035241	0.0903	0.1443

1985∼1990 - 1995∼2000				

Total Migration	0.15402	-0.07095	0.3370	0.0023
In-Migration	0.02560	-0.03412	0.4495	0.0002
Out-Migration	-0.00720	-0.00442	0.0067	0.6963
Intra-Migration	0.02185	-0.01147	0.0114	0.6112

**Table 3.** Regression analysis between change in NDVI, rate of change in gross domestic production (GDP), changes in mean annual temperature and total annual precipitation from 1982∼1987 to 1995∼2000: ΔV_31_=b_0_+b_1_X, where X is ΔGDP_31_, ΔT_31_, ΔP_31_ and ΔM_31_*, respectively. Here ΔM_31_* indicates the effect of changes in temperature and precipitation on ΔV_31_ is removed before it is regressed with ΔM_31_.

X	b_0_	b_1_	R^2^		P-value
ΔGDP31	0.38024	-0.03307	0.2542	0.0102	
ΔT_31_	0.11244	0.10218	0.4992	0.0001	
ΔP_31_	0.13597	-0.00169	0.2962	0.0049	
ΔM_31_*	0.197511	-0.05706	0.5572	0.0000	
